# Length of stay and associated costs of obesity related hospital admissions in Ireland

**DOI:** 10.1186/1472-6963-8-88

**Published:** 2008-04-22

**Authors:** Akke Vellinga, Diarmuid O'Donovan, Davida De La Harpe

**Affiliations:** 1 Department of Public Health, Health Service Executive West, Galway, Ireland; 2 Department of Health Promotion, National University Ireland, Galway, Ireland; 3 Population Health, Health Intelligence, Health Service Executive, Dublin, Ireland

## Abstract

**Background:**

Obesity is the cause of other chronic diseases, psychological problems, obesity shortens the lifespan and puts strain on health systems. The risk associated with childhood obesity in particular, which will accelerate the development of adult morbidity and mortality, has been identified as an emerging public health problem.

**Methods:**

To estimate the length of stay and associated hospital costs for obesity related illnesses a cost of illness study was set up. All discharges from all acute hospitals in the Republic of Ireland from 1997 to 2004 with a principal or secondary diagnostic code for obesity for all children from 6 to 18 years of age and for adults were collected.

A discharge frequency was calculated by dividing obesity related discharges by the total number of diagnoses (principal and secondary) for each year. The hospital costs related to obesity was calculated based on the total number of days care.

**Results:**

The discharge frequency of obesity related conditions increased from 1.14 in 1997 to 1.49 in 2004 for adults and from 0.81 to 1.37 for children. The relative length of stay (number of days in care for obesity related conditions per 1000 days of hospital care given) increased from 1.47 in 1997 to 4.16 in 2004 for children and from 3.68 in 1997 to 6.74 in 2004 for adults.

Based on the 2001 figures for cost per inpatient bed day, the annual hospital cost was calculated to be 4.4 Euromillion in 1997, increasing to 13.3 Euromillion in 2004. At a 20% variable hospital cost the cost ranges from 0.9 Euromillion in 1997 to 2.7 Euromillion in 2004; a 200% increase.

**Conclusion:**

The annual increase in the proportion of hospital discharges related to obesity is alarming. This increase is related to a significant increase in economic costs. This paper emphasises the need for action at an early stage of life. Health promotion and primary prevention of obesity should be high on the political agenda.

## Background

The prevalence of obesity and overweight has increased dramatically over the past decades and researchers are only gradually becoming aware of the gravity of the risk posed [1,2]. In particular, the risk associated with childhood obesity, which will accelerate the development of adult morbidity and mortality, has been identified as an emerging public health problem [3]. The possibility that the current generation of children could suffer greater illness or experience a shorter lifespan than that of their parents has been suggested to be possible [4].

Obesity is the cause of serious chronic disease. Health consequences of obesity include diabetes mellitus, asthma, sleep apnoea, gall bladder disease and a range of cancers [5]. Obesity is known to reduce quality of life and impact on psychological problems [2,4].

Overweight is a term principally used to describe a body mass index over the 95^th ^percentile by age and gender [6]. Rates of obesity vary between 10–20% for men and 10–25% of women in different countries [7]. In the Republic of Ireland a survey from 2001 found 39% of adults to be overweight and 18% obese [8].

Data for Irish children are scarce but a recent survey in one county (Mayo, West of Ireland) where public health nurses weighed and measured all school children aged five to seven showed that 27% were overweight or obese with overall 7% of the six year olds obese [9]. Data from cross national surveys in which weight and length were self reported indicate that 13.7% of the Irish children between 10 and 16 years of age were overweight [10].

Many ubiquitous ties to a variety of health conditions, population level approaches to estimations of the total cost burden range from 2% of the national health care budget for most industrialised countries to up to 5–7% in the United States [11]. According to the report of the Irish National Task force on Obesity (2005), estimated inpatient cost of obesity as primary diagnosis in 2003 were just over €150,000 and the proportion of diagnosis attributable to obesity was estimated to be just under €30 million based on an estimation of the relative risk ratio [12]. An approach used by Wang et al (2002) to calculate the economic burden of obesity using primary and secondary discharge codes showed it to be a more precise to estimate obesity related hospital cost which include co-morbidities as well as allow for comparisons between years [13].

Following Wang's example, this paper analyses the length of stay and hospital costs associated with obesity and related conditions for adults aged 18 and older and for children aged between 6 and 17 inclusive. To analyse the change in obesity related hospital stays data were extracted from 1997 to 2004.

## Methods

### Data source

The Hospital In-Patient Enquiry (HIPE) is the principal source of national data on discharges from all acute hospitals in the Republic of Ireland [14]. Hospital chart information is entered by trained HIPE coders into a computer. Each HIPE record represents one episode of care. Over 60 acute public hospitals participate in HIPE reporting on around 900,000 admissions annually. Over the years different adaptations of the database have been introduced. Until 2001, up to 6 diagnoses were included for each discharge, and from 2002 to 2004 up to 10 diagnoses have been included. The coding system used is the International Classification of Diseases, Ninth Revision, Clinical Modification (ICD-9-CM). Discharges rather then admissions are used as HIPE is a hospital based discharge database; patients only enter the system once they leave the hospital. Since there are no unique identifiers available, multiple discharges are possible for one person.

All discharges for obesity (ICD-9 code 278) were included. Obesity is described by the educational annotation of ICD-9-CM as an abnormal amount of fat on the body irrespective of BMI measurements and according to the consultants' judgement. The diagnostic code listed first was used as the principal diagnosis, and subsequent diagnostic codes (second through sixth or tenth) were used as secondary diagnoses.

The codes for 'symptoms and signs of ill defined conditions' (ICD 780–799) are assigned for symptoms and signs which are not directly linked to a specific disease.

### Analyses

The obesity related hospital discharges from 1997 to 2004 were identified and extracted for all discharges for children from 6 to 18 years (≥ 6 and <18) of age and for adults (≥ 18). All principal (first listed diagnostic code) and secondary (second and higher codes) diagnoses were used. A discharge frequency was calculated by dividing obesity related discharges by the total number of diagnoses (principal and secondary) for each year as this will allow for the change in registration practice over the years. All discharges with obesity as first code were considered for obesity, obesity related discharges were defined as having a secondary code of obesity.

Length of stay was recorded for each hospital stay related to obesity. The average hospital cost per day was derived from the report of the Commission on Financial Management and Control systems in Health Service [15]. The 2001 average cost per inpatient bed day is baseline and the Consumer Price Index (Base 2001) for services is used to derive the other years [16]. A guide for relative versus fixed costs of inpatient care was chosen to be at 20% versus 80% [17]. Fixed costs include capital expenditures, employee salaries and benefits, building maintenance, and utilities. Variable costs include health care worker supplies, patient care supplies, diagnostic and therapeutic supplies and medication. Data analysis was performed with SPSS for Windows (Chicago Ltd Version 14.0).

## Results

The total number of principal diagnoses of obesity increased from 680,238 in 1997 to 984,137 in 2004, an increase of 45% (Table 1). The number of principal diagnoses for adults went up from 551,931 to 843,839 over the same period, an increase of 53%. For children the numbers of principal diagnoses decreased over the same period from 67,086 in 1997 to 63,253, or with 5.7%. The overall total number of diagnoses are not compared due to changes in reporting systems in 2003.

**Table 1:** Obesity related discharges and length of stay from 1997 to 2004 for adults and children.

	1997		1998		1999		2000		2001		2002		2003		2004	
	Adult	Child	Adult	Child	Adult	Child	Adult	Child	Adult	Child	Adult	Child	Adult	Child	Adult	Child
**Obesity Discharges**																
Principal	38	29	31	12	27	13	30	33	32	18	36	28	38	31	64	42
Secondary	1498	64	2361	96	2231	102	2522	113	2795	116	3824	158	3810	154	3549	135
Total	1536	93	2392	108	2258	115	2552	146	2827	134	3860	186	3848	185	3613	177
**Length of Stay**																
Average	7.2	2.9	6.8	4.3	6.4	3.5	6.0	2.8	7.7	3.1	7.0	2.3	7.4	2.4	6.8	3.3
Median	5	2	4	2	4	2	4	2	4	1	4	1	3	1	4	1
Principal	187	114	182	94	205	28	192	80	222	78	296	58	267	74	353	187
Secondary diagnoses	10930	157	16062	371	14222	378	15019	332	21543	344	26657	380	28350	368	24190	402

Total	11117	271	16244	465	14427	406	15211	412	21765	422	26953	438	28617	442	24543	589

The discharge frequency for obesity related conditions increased from 1.14 in 1997 to 1.49 in 2004 for adults and from 0.81 to 1.37 for children (Table 2).

**Table 2:** Total number of discharges, total length of stay, discharge frequency and relative length of stay from 1997 to 2004 for adults and children.

	1997		1998		1999		2000		2001		2002		2003		2004	
	Adult	Child	Adult	Child	Adult	Child	Adult	Child	Adult	Child	Adult	Child	Adult	Child	Adult	Child
Total discharges (10^3^)	552	67	568	65	632	63	667	63	724	64	760	60	800	63	844	63
Total diagnoses (10^3^)	1351	114	1441	114	1617	114	1726	114	1880	119	2323	119	2275	128	2425	130
**Length of Stay**																
Average	5.5	2.7	5.3	2.6	5.0	2.6	4.9	2.5	4.7	2.5	4.5	2.4	4.3	2.3	4.3	2.2
Total (10^3^)	3022	184	3037	172	3159	166	3257	158	3406	157	3448	142	3481	143	3644	142
Discharge frequency*																
	1.14	0.81	1.66	0.94	1.40	1.01	1.48	1.29	1.50	1.13	1.66	1.57	1.70	1.45	1.49	1.37
Relative length of stay**																
	3.7	1.5	5.3	2.7	4.6	2.4	4.7	2.6	6.4	2.7	7.8	3.1	8.2	3.1	6.7	4.2

*per 1000 diagnoses

**per 1000 days of hospital care

When obesity was listed as a secondary diagnosis in adults, the most frequent principal code was ICD 786; symptoms involving respiratory system and other chest symptoms (8.5%, ranging from 6.5 in 1999 to 9.4 in 2002 and 2003). This diagnosis includes dyspnoea, hyperventilation, wheezing and unspecified chest pain. Other frequent principal codes with obesity as a secondary diagnosis, were ICD 250; diabetes (3.6%, ranging from 1.6 in 2004 to 4.1 in 1997) and ICD 491; chronic bronchitis (3.3%, ranging from 2.7 in 2003 to 4.4 in 1999). Ischemic heart diseases were a frequent diagnosis, with codes ICD 410; Acute myocardial infarction (2.7%, ranging from 2.4% in 2002 to 3.3% in 2000), ICD 411; other acute and sub-acute forms of ischemic heart disease (3.1%, ranging from 2.3 in 2004 to 3.6 in 1998) and ICD 414; other forms of chronic ischemic heart disease (6.1%, ranging from 4.6 in 2000 to 7.6 in 1998). Other frequent circulatory problems were ICD 427; cardiac dysrhythmias (2.8, ranging from 2.4% in 1998 to 3.3% in 1998 and 1999), ICD 428; heart failure (2.8%, ranging from 2.4 in 1998 to 3.2% in 2002) and ICD 401; essential hypertension (2.7%, ranging from 1.6 in 2004 to 3.5% in 1999).

For children the most frequent principal morbidities with obesity as secondary diagnosis were asthma (ICD 493, 7.7%), symptoms, signs and ill defined conditions involving abdomen and pelvis (ICD 789, 5.4%), Epilepsy (ICD 345, 4.5%), functional digestive disorders (ICD 564, 4.1%), general symptoms, signs and ill defined conditions (ICD 780, 3.8%) and other endocrine disorders, not specified (ICD 259, 3.4%).

**Figure 1:**
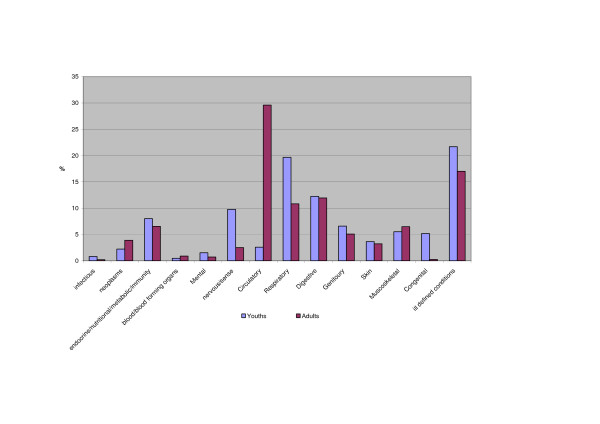
Principal diagnoses by disease group for discharges where obesity was listed as secondary diagnosis for adults and children.

Primary diagnoses aggregated into disease categories when obesity is recorded as a secondary diagnosis, are shown in Figure 1. The main disease group in adults constituting one third of the principal diagnoses, is circulatory disease. For children this group is small and most morbidities are related to respiratory diseases (14–26% of the principal diagnoses).

The disease group 'symptoms and signs and ill defined conditions' included convulsions, dizziness, sleep disturbances, lack of normal physiological development, headache, and abdominal pain for children. All these symptoms and additional diagnoses of syncope and collapse, epistaxis, palpitations and chest pain were seen in adults.

To estimate the hospital cost related to obesity the number of days of care were analysed. The total number of days care were calculated per year as well as the total length of stay for obesity and obesity related conditions.

Table 3 shows the length of stay associated with obesity for adults and children. The overall LOS is significantly (p = 0.000) higher for adults (median 4.0, mean 6.9) compared to children (median 3.0, mean 3.0). Differences in LOS from year to year do not fluctuate much for adults but decline from a median of 2 to 1 from 2000 for children. The relative length of stay (number of days in care for obesity related conditions per 1000 days of hospital care given) increased over time from 1.5 in 1997 to 4.2 in 2004 for children and from 3.7 in 1997 to 6.7 in 2004 for adults.

**Table 3:** Obesity related length of stay and hospital costs from 1997 to 2004 among adults and children

	1997	1998	1999	2000	2001	2002	2003	2004
Total number of hospital bed days for obesity related illness								
	11388	16709	14833	15623	22187	27391	29059	25132
								
Cost per inpatient bed day (base year 2001)								
	388	401	407	432	**477**	497	520	538
								
Total hospital cost (Euromillions, fixed and variable)								
	4.4	6.7	6.0	6.7	10.6	13.6	15.1	13.5
								
Variable costs (Euromillions)								
	0.9	1.3	1.2	1.3	2.1	2.7	3.0	2.7

**Figure 2:**
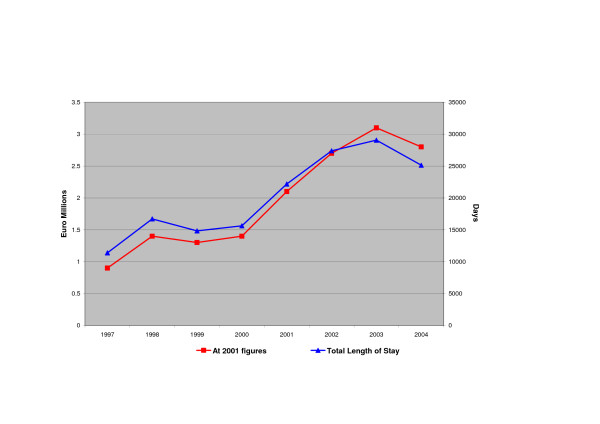
Variable hospital costs and total length of stay from 1997 to 2004 for obesity related conditions.

The total length of stay and the associated cost are shown in Figure 2. Based on the 2001 figures for cost per inpatient bed day, the annual hospital cost was calculated to be 4.4 Euro million in 1997, increasing to 13.3 Euro million in 2004. At a 20% variable hospital cost the cost ranges from 0.9 Euromillion in 1997 to 2.7 Euromillion in 2004; a 200% increase.

## Discussion

The increase in hospital discharges related to obesity increased dramatically during the study period, especially for children. Even though the numbers are small, the increases are substantial and consistent over time. This increase comes with a significant growth in economic costs.

The increase reflects the higher proportion of hospitalisations for obesity. The median stay per obesity related condition did not show consistent changes over the years indicating that the severity of the cases did not change over the years, for children the median stay even declined. The increase in hospitalisations is therefore an increase in the number of hospitalisations for obesity related conditions and not their severity. However, the fact that obesity is coded and the person is hospitalised, implies a severe case.

The code for obesity was most often entered as secondary diagnosis which suggests that the estimates made in this paper are very conservative. Firstly, a secondary diagnosis will less often be coded and treatment will usually be for a related condition rather then obesity. Secondly, the dependency of the analysis on consultants to report obesity, especially to include it as secondary code, will lead to underreporting. As the number of secondary diagnoses increased from 5 to 9 the chance to include obesity as a secondary code also increased and might explain the jump between 2000 and 2001 in the total length of stay (Figure 2). However, the increase continues on over the following years emphasising the underlying increasing trend in obesity related conditions.

The differences in co-morbidities in the younger age groups compared to the adults lies mainly in the increase of cardiovascular diseases. Asthma and other respiratory diseases in children are well known co-morbidities of obesity. It is not easy to distinguish whether the respiratory complaints result in obesity or the obesity results in respiratory complaints, but the link is clearly physical activity [18]. Overweight and obesity are associated with a dose-dependent increase in the odds of incident asthma in both men and women, suggesting asthma incidence could be reduced by interventions targeting overweight and obesity. It has been shown that asthma was identified as a barrier to exercise by parents and children [19]. Strategies to promote exercise within paediatric asthma care are needed to protect both mental and physical health [20,21]. Implementing interventions in childhood can also result in more long term improvements by its beneficial effects on circulatory disease in adults.

The hospital costs related to obesity were calculated at 13.3 Euromillions in 2004 and 15.1 Euromillions in 2003. In comparison, the report of the National Taskforce on Obesity calculated the hospital related cost to be €150,000 in 2003 where obesity was listed as the primary diagnosis and €30 million estimates were made on the basis of selected diagnosis attributable to obesity [12]. The presented calculation gives a more accurate estimate of hospital costs and emphasises again the need for better recording of all diagnosis. The HIPE database has introduced a new coding scheme (ICD-10-AM) in 2005 which allows up to 20 diagnoses to be entered as well as a number of personal details including weight. This will improve future estimates of obesity related costs as well as allow for the analysis of dose-dependent increase in costs.

This study has some limitations. While the use of a national hospital database has great potential, there are obvious limitations to the same database [22]. Due to the lack of personal identifiers in the HIPE database, it is impossible to estimate prevalence from the discharge frequency. Secondly, the dependence on consultants to record obesity is a shortcoming. In a retrospective medical record review of health visits of children to a hospital in Pennsylvania (US) the diagnosis of obesity was not made for 53% of the children despite fulfilling the criteria according to their records [23].

The aim of the study was in the first place to estimate the length of stay and associated cost, related to obesity. This cost of illness study suggests that these costs could potentially be saved if obesity would be eradicated [24]. Indeed, unlike many other diseases, obesity is a condition for which this 'illusion' of eradication can potentially be true [21]. Primary prevention by health promotion campaigns and secondary by mental and dietary treatment can ideally abolish the hospital costs obesity inflicts on society [20,25]. However, even if preventive measures against obesity were successful immediately, there would still be an epidemic of its consequences as so many young people are already in the clinically 'latent' phase. Obesity, unlike many other chronic diseases, can be treated successfully and the most effective strategy to improve the health of a population may be to prioritise and provide incentives for the management of obesity. Enhanced awareness both on reporting on obesity as well as the development and monitoring of interventions were the initial reason for this cost of illness analysis.

## Conclusion

The annual increase in hospital discharges and the cost of hospital care is alarming. The rising disease burden associated with obesity in children in particular indicates the increasing future pressure of obesity related diseases at an older age. This emphasises again the need for action at an early stage of life. The clear link between obesity in children and its progression into adulthood predicts an even bigger increase in the long term. Health promotion and primary prevention of obesity should be high on the political agenda.

## Competing interests

This research was funded by the Health Service Executive through a grant to the National University Ireland, Galway.

## Authors' contributions

AV conceived of the study, performed the statistical analysis and drafted the manuscript. DoD and DdlH helped to draft the manuscript. All authors read and approved the final manuscript.

## Pre-publication history

The pre-publication history for this paper can be accessed here:


